# Supplementary material for: Sequence Variation of Rare Outer Membrane Protein β-Barrel Domains in Clinical Strains Provides Insights into the Evolution of Treponema pallidum subsp. pallidum, the Syphilis Spirochete
Source: mBio. 2018 Jun 12;9(3):e01006-18. doi: 10.1128/mBio.01006-18 (PMC6016234; doi:10.1128/mBio.01006-18)
Supplement: TABLE S1 [file mbo003183920st1.docx]

**Table S1. Summary of available geographic, clinical, and demographic data for clinical samples**

| **Patient** | **City** | **Year of isolation** | **Gender** | **Syphilis**  **stage** | **Sample type** | **HIV**  **status** | **Race/**  **Ethnicity** | **Sexual**  **Orientation** |
| --- | --- | --- | --- | --- | --- | --- | --- | --- |
| Cali_77 | Cali | 2009 | F | Secondary | Skin Biopsy | Negative | Mestizo | Heterosexual |
| Cali_84 | Cali | 2009 | M | Secondary | Skin Biopsy | Negative | Black | Heterosexual |
| Cali_101 | Cali | 2009 | M | Secondary | Skin Biopsy | Negative | Mestizo | Heterosexual |
| Cali_103 | Cali | 2009 | F | Secondary | Skin Biopsy | Negative | Black | Heterosexual |
| Cali_123 | Cali | 2010 | F | Secondary | Skin Biopsy | Negative | Black | Heterosexual |
| Cali_127 | Cali | 2011 | M | Secondary | Skin Biopsy | Negative | Black | Heterosexual |
| Cali_130 | Cali | 2011 | F | Secondary | Skin Biopsy | Negative | Black | Heterosexual |
| Cali_133 | Cali | 2013 | F | Secondary | Skin Biopsy | Negative | Black | Heterosexual |
| Cali_143 | Cali | 2013 | F | Secondary | Skin Biopsy | Negative | Black | Heterosexual |
| Cali_145 | Cali | 2013 | F | Secondary | Skin Biopsy | Negative | Mestizo | Heterosexual |
| Cali_146 | Cali | 2014 | M | Secondary | Skin Biopsy | Negative | Mestizo | MSM^1^ |
| Cali_151 | Cali | 2014 | M | Secondary | Skin Biopsy | Negative | Mestizo | Heterosexual |
| Cali_153 | Cali | 2014 | M | Secondary | Skin Biopsy | Negative | Mestizo | Heterosexual |
| Cali_156 | Cali | 2014 | F | Secondary | Skin Biopsy | Negative | Mestizo | Heterosexual |
| Cali_164 | Cali | 2014 | M | Secondary | Skin Biopsy | Negative | Mestizo | Heterosexual |
| Cali_167 | Cali | 2014 | M | Secondary | Skin Biopsy | Negative | Black | Heterosexual |
| SF_6 | San Francisco | 2004 | M | Primary | Chancre Swab | Negative | Hispanic | Heterosexual |
| SF_7 | San Francisco | 2004 | M | Primary | Chancre Swab | Negative | Hispanic | MSM |
| SF_40 | San Francisco | 2006 | M | Primary | Chancre Swab | Negative | Asian | Heterosexual |
| SF_46 | San Francisco | 2006 | M | Primary | Chancre Swab | Negative | Caucasian | MSM |
| SF_50 | San Francisco | 2006 | M | Primary | Chancre Swab | Negative | Black | Bisexual |
| SF_58 | San Francisco | 2007 | M | Primary | Chancre Swab | Negative | Hispanic | Bisexual |
| CZ_177zB | Brno | 2013 | M | Primary | Skin swab | Unknown | Caucasian | Unknown |
| CZ_178zB | Prague | 2013 | M | Secondary | Skin swab | Unknown | Caucasian | Unknown |
| CZ_190Z | Prague | 2014 | M | Secondary | Genital lesion swab | Negative | Caucasian | Unknown |
| CZ_192Z | Brno | 2014 | M | Primary | Skin swab | Positive | Caucasian | Unknown |
| CZ_3218 | Brno | 2014 | M | Primary | Genital lesion swab | Unknown | Caucasian | Unknown |
| CZ_351 | Prague | 2016 | M | Primary | Genital lesion swab | Unknown | Caucasian | Unknown |
| CZ_4535 | Prague | 2015 | M | Secondary | Lesion swab | Unknown | Caucasian | Unknown |
| CZ_PP1979B | Brno | 2014 | M | Primary | Perianal lesion swab | Negative | Caucasian | MSM |
| CZ_S1120 | Brno | 2015 | M | Primary | Genital lesion swab | Unknown | Caucasian | Unknown |

^1^MSM, Men who have sex with men

^2^San Francisco
