# Supplementary figures and images for: Sequence Variation of Rare Outer Membrane Protein β-Barrel Domains in Clinical Strains Provides Insights into the Evolution of Treponema pallidum subsp. pallidum, the Syphilis Spirochete
Source: mBio. 2018 Jun 12;9(3):e01006-18. doi: 10.1128/mBio.01006-18 (PMC6016234; doi:10.1128/mBio.01006-18)

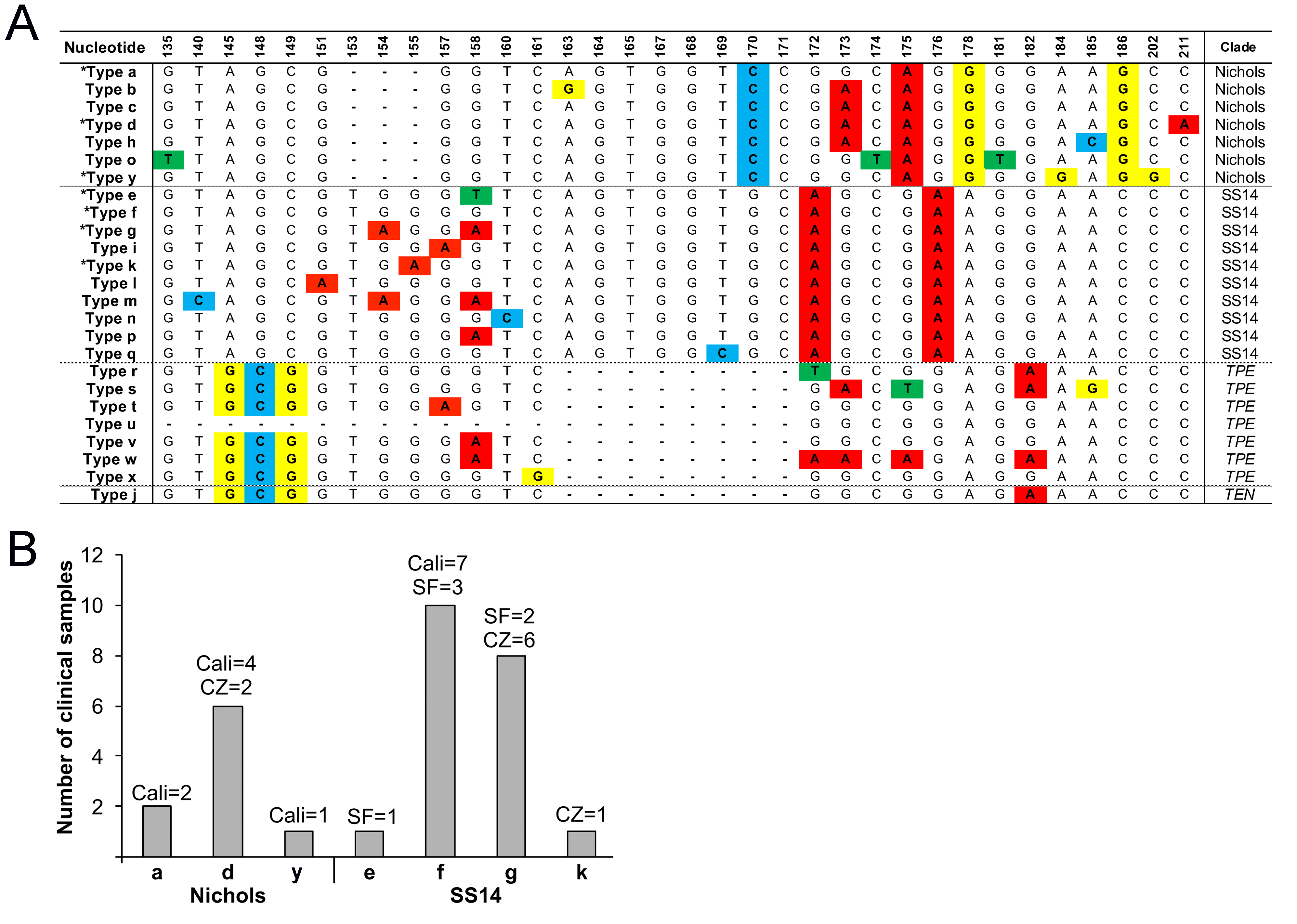

Supplement: FIG S1 [file mbo003183920sf1.tif]

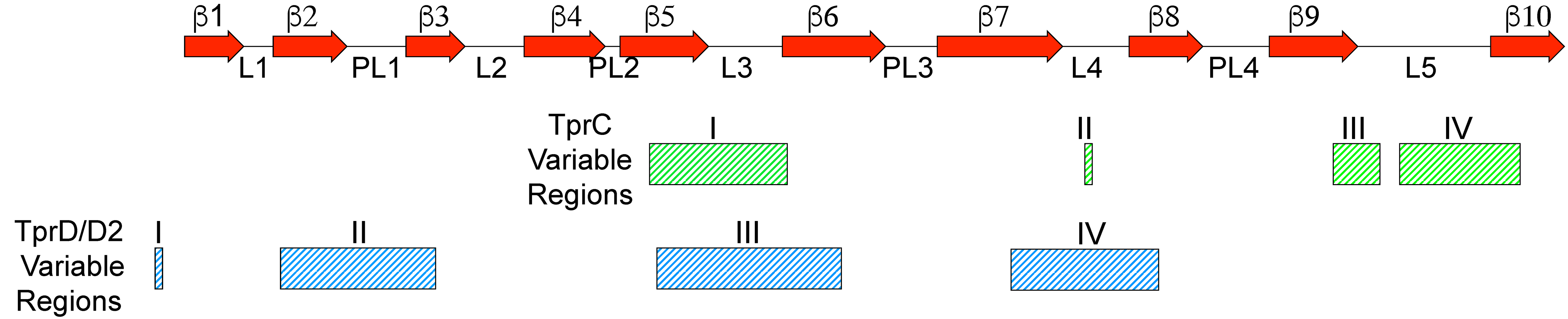

Supplement: FIG S5 [file mbo003183920sf5.tif]
