# Supplementary material for: Sequence Variation of Rare Outer Membrane Protein β-Barrel Domains in Clinical Strains Provides Insights into the Evolution of Treponema pallidum subsp. pallidum, the Syphilis Spirochete
Source: mBio. 2018 Jun 12;9(3):e01006-18. doi: 10.1128/mBio.01006-18 (PMC6016234; doi:10.1128/mBio.01006-18)
Supplement: FIG S2 [file mbo003183920sf2.docx]

                    10        20        30        40        50        60        70        80        90     100

Nichols GTGGGCAGGCAGGTGATGCAAGCGGGGGTACTTGCGGGCATGGTATGTGCTGCTTCTGGTTATGCAGGCGTACTCACTCCGCAGGTCAGTGGCACAGCCC

Mexico A   GTGGGCAGGCAGGTGATGCAAGCGGGGGTACTTGCGGGCATGGTATGTGCTGCTTCTGGTTATGCAGGCGTACTCACTCCGCAGGTCAGTGGCACAGCCC

PT_SIF     GTGGGCAGGCAGGTGATGCAAGCGGGGGTACTTGCGGGCATGGTATGTGCTGCTTCTGGTTATGCAGGCGTACTCACTCCGCAGGTCAGTGGCACAGCCC

Sea81-4    GTGGGCAGGCAGGTGATGCAAGCGGGGGTACTTGCGGGCATGGTATGTGCTGCTTCTGGTTATGCAGGCGTACTCACTCCGCAGGTCAGTGGCACAGCCC

SS14       GTGGGCAGGCAGGTGATGCAAGCGGGGGTACTTGCGGGCATGGTATGTGCTGCTTCTGGTTATGCAGGCGTACTCACTCCGCAGGTCAGTGGCACAGCCC

          ****************************************************************************************************

                  110       120       130       140       150       160       170       180       190      200

Nichols AGCTCCAGTGGGGCATTGCGTTCCAGAAGAATCCACGCACTGGCCCGGGCAAGCACACCCATGGGTTTCGCACTACCAATAGTCTGACTATTTCCCTGCC

Mexico A   AGCTCCAGTGGGGCATTGCGTTCCAGAAGAATCCACGCACTGGCCCGGGCAAGCACACCCATGGGTTTCGCACTACCAATAGTCTGACTATTTCCCTGCC

PT_SIF     AGCTCCAGTGGGGCATTGCGTTCCAGAAGAATCCACGCACTGGCCCGGGCAAGCACACCCATGGGTTTCGCACTACCAATAGTCTGACTATTTCCCTGCC

Sea81-4    AGCTCCAGTGGGGCATTGCGTTCCAGAAGAATCCACGCACTGGCCCGGGCAAGCACACCCATGGGTTTCGCACTACCAATAGTCTGACTATTTCCCTGCC

SS14       AGCTCCAGTGGGGCATTGCGTTCCAGAAGAATCCACGCACTGGCCCGGGCAAGCACACCCATGGGTTTCGCACTACCAATAGTCTGACTATTTCCCTGCC

           ****************************************************************************************************

                  210       220       230       240       250       260       270       280       290       300

Nichols GTTGGTGTCAAAGCACACCCACACCCGCCGAGGGGAGGCACGCTCAGGG**GTGTGGGCACAGCTGCAGCTGAAGGACCTGGCAGTAGAGCTTGCGTCTTCT**

Mexico A   GTTGGTGTCAAAGCACACCCACACCCGCCGAGGGGAGGCACGCTCAGGG**GTGTGGGCACAGCTGCAGCTGAAGGACCTGGCAGTAGAGCTTGCGTCTTCT**

PT_SIF     GTTGGTGTCAAAGCACACCCACACCCGCCGAGGGGAGGCACGCTCAGGG**GTGTGGGCACAGCTGCAGCTGAAGGACCTGGCAGTAGAGCTTGCGTCTTCT**

Sea81-4    GTTGGTGTCAAAGCACACCCACACCCGCCGAGGGGAGGCACGCTCAGGG**GTGTGGGCACAGCTGCAGCTGAAGGACCTGGCAGTAGAGCTTGCGTCTTCT**

SS14       GTTGGTGTCAAAGCACACCCACACCCGCCGAGGGGAGGCACGCTCAGGG**GTGTGGGCACAGCTGCAGCTGAAGGACCTGGCAGTAGAGCTTGCGTCTTCT**

           ****************************************************************************************************

                 310       320       330       340       350       360       370       380       390    400

Nichols **AAAAGCTCAACGGCCCTGTCCTTTACCAAACCTACCGCTTCCTTCCAGGCAACCCTGCACTGTTATGGGGCCTACCTGACAGTGGGTACCAGTCCTTCCT**

Mexico A   **AAAAGCTCAACGGCCCTGTCCTTTACCAAACCTACCGCTTCCTTCCAGGCAACCCTGCACTGTTATGGGGCCTACCTGACAGTGGGTACCAGTCCTTCCT**

PT_SIF     **AAAAGCTCAACGGCCCTGTCCTTTACCAAACCTACCGCTTCCTTCCAGGCAACCCTGCACTGTTATGGGGCCTACCTGACAGTGGGTACCAGTCCTTCCT**

Sea81-4    **AAAAGCTCAACGGCCCTGTCCTTTACCAAACCTACCGCTTCCTTCCAGGCAACCCTGCACTGTTATGGGGCCTACCTGACAGTGGGTACCAGTCCTTCCT**

SS14       **AAAAGCTCAACGGCCCTGTCCTTTACCAAACCTACCGCTTCCTTCCAGGCAACCCTGCACTGTTATGGGGCCTACCTGACAGTGGGTACCAGTCCTTCCT**

           ****************************************************************************************************

                 410       420       430       440       450       460       470       480       490       500

Nichols **GTGTGGTTAACTTTGCCCAGCTGTGGAAACCCTTTGTCACCCGTGCCTATTCAGAAAAGGACACTCGCTATGCCCCTGGTTTCTCCGGCTCCGGGGCAAA**

Mexico A   **GTGTGGTTAACTTTGCCCAGCTGTGGAAACCCTTTGTCACCCGTGCCTATTCAGAAAAGGACACTCGCTATGCCCCTGGTTTCTCCGGCTCCGGGGCAAA**

PT_SIF     **GTGTGGTTAACTTTGCCCAGCTGTGGAAACCCTTTGTCACCCGTGCCTATTCAGAAAAGGACACTCGCTATGCCCCTGGTTTCTCCGGCTCCGGGGCAAA**

Sea81-4    **GTGTGGTTAACTTTGCCCAGCTGTGGAAACCCTTTGTCACCCGTGCCTATTCAGAAAAGGACACTCGCTATGCCCCTGGTTTCTCCGGCTCCGGGGCAAA**

SS14       **GTGTGGTTAACTTTGCCCAGCTGTGGAAACCCTTTGTCACCCGTGCCTATTCAGAAAAGGACACTCGCTATGCCCCTGGTTTCTCCGGCTCCGGGGCAAA**

           ****************************************************************************************************

                  510       520       530       540       550       560       570       580       590       600

Nichols **ACTCGGCTACCAGGCCCACAATGTGGGAAACAGCGGAGTAGATGTGGACATCGGTTTCCTCTCCTTCCTTTCCAATGGTGCCTGGGATAGTACTGACACC**

Mexico A  **ACTCGGCTACCAGGCCCACAATGTGGGAAACAGCGGAGTAGATGTGGACATCGGTTTCCTCTCCTTCCTTTCCAATGGTGCCTGGGATAGTACTGACACC**

PT_SIF     **ACTCGGCTACCAGGCCCACAATGTGGGAAACAGCGGAGTAGATGTGGACATCGGTTTCCTCTCCTTCCTTTCCAATGGTGCCTGGGATAGTACTGACACC**

Sea81-4    **ACTCGGCTACCAGGCCCACAATGTGGGAAACAGCGGAGTAGATGTGGACATCGGTTTCCTCTCCTTCCTTTCCAATGGTGCCTGGGATAGTACTGACACC**

SS14       **ACTCGGCTACCAGGCCCACAATGTGGGAAACAGCGGAGTAGATGTGGACATCGGTTTCCTCTCCTTCCTTTCCAATGGTGCCTGGGATAGTACTGACACC**

           ****************************************************************************************************

                  610       620       630       640       650       660       670       680       690       700

Nichols **ACGCACAGCAAGTATGGCTTCGGGGCCGATGCAACGCTTTCCTATGGCGTCGACCGTCAGCGGCTGCTTACGTTGGAGCTGGCAGGGAATGCCACACTGG**

Mexico A   **ACGCACAGCAAGTATGGCTTCGGGGCCGATGCAACGCTTTCCTATGGCGTCGACCGTCAGCGGCTGCTTACGTTGGAGCTGGCAGGGAATGCCACACTGG**

PT_SIF     **ACGCACAGCAAGTATGGCTTCGGGGCCGATGCAACGCTTTCCTATGGCGTCGACCGTCAGCGGCTGCTTACGTTGGAGCTGGCAGGGAATGCCACACTGG**

Sea81-4    **ACGCACAGCAAGTATGGCTTCGGGGCCGATGCAACGCTTTCCTATGGCGTCGACCGTCAGCGGCTGCTTACGTTGGAGCTGGCAGGGAATGCCACACTGG**

SS14       **ACGCACAGCAAGTATGGCTTCGGGGCCGATGCAACGCTTTCCTATGGCGTCGACCGTCAGCGGCTGCTTACGTTGGAGCTGGCAGGGAATGCCACACTGG**

           ****************************************************************************************************

MOSP^N^ variable region

                   710       720       730       740       750       760       770       780       790       800

Nichols **ACCAGAACTACGTTAAGGGTACCGAAGACTCCAAGAACGAAAACAAAACAGCACTCCTGTGGGGAGTAGGAGGCCGACTCACCCTCGAACCAGGCGCCGG**

Mexico A   **AGCAGCACTACCGTAAGGGTACCGAAGACTCCACGAACGAAAACAAAACAGCACTCCTGTGGGGAGTAGGAGGCCGACTCACCCTCGAACCAGGCGCCGG**

PT_SIF     **ACCAGAACTACGTTAAGGGTACCGAAGACTCCAAGAACGAAAACAAAACAGCACTCCTGTGGGGAGTAGGAGGCCGACTCACCCTCGAACCAGGCGCCGG**

Sea81-4    **ACCAGAACTACGTTAAGGGTACCGAAGACTCCAAGAACGAAAACAAAACAGCACTCCTGTGGGGAGTAGGAGGCCGACTCACCCTCGAACCAGGCGCCGG**

SS14       **ACCAGAACTACGTTAAGGGTACCGAAGACTCCAAGAACGAAAACAAAACAGCACTCCTGTGGGGAGTAGGAGGCCGACTCACCCTCGAACCAGGCGCCGG**

           * *** *****  ******************** ******************************************************************

                   810       820       830       840       850       860       870       880       890       900

Nichols **CTTCCGCTTCTCCTTCGCCCTCGACGCCGGTAACCAACACCAGAGTAACGCACATGCTCAGACCCAAGAGAGAGCTATCCTCAAAGCAAGGGAAGTGTTT**

Mexico A   **CTTCCGCTTCTCCTTCGCCCTCGACGCCGGTAACCAACACCAGAGTAACGCACATGCTCAGACCCAAGAGAGAGCTATCCTCAAAGCAAGGGAAGTGTTT**

PT_SIF     **CTTCCGCTTCTCCTTCGCCCTCGACGCCGGTAACCAACACCAGAGTAACGCACATGCTCAGACCCAAGAGAGAGCTATCCTCAAAGCAAGGGAAGTGTTT**

Sea81-4    **CTTCCGCTTCTCCTTCGCCCTCGACGCCGGTAACCAACACCAGAGTAACGCACATGCTCAGACCCAAGAGAGAGCTATCCTCAAAGCAAGGGAAGTGTTT**

SS14       **CTTCCGCTTCTCCTTCGCCCTCGACGCCGGTAACCAACACCAGAGTAACGCACATGCTCAGACCCAAGAGAGAGCTATCCTCAAAGCAAGGGAAGTGTTT**

           ****************************************************************************************************

                   910       920       930       940       950       960       970       980       990      1000

Nichols **AGACGGGTGGAGGGGAAACTCGTGCAGAACCTTCCCAATATCATGATGCCACCAGGAATCACCGAACAAACCACTCTCATAGAGATGGTAGGACTTGCTG**

Mexico A   **AGACGGGTGGAGGGGAAACTCGTGCAGAACCTTCCCAATATCATGATGCCACCAGGAATCACCGAACAAACCACTCTCATAGAGATGGTAGGACTTGCTG**

PT_SIF     **AGACGGGTGGAGGGGAAACTCGTGCAGAACCTTCCCAATATCATGATGCCACCAGGAATCACCGAACAAACCACTCTCATAGAGATGGTAGGACTTGCTG**

Sea81-4    **AGACGGGTGGAGGGGAAACTCGTGCAGAACCTTCCCAATATCATGATGCCACCAGGAATCACCGAACAAACCACTCTCATAGAGATGGTAGGACTTGCTG**

SS14       **AGACGGGTGGAGGGGAAACTCGTGCAGAACCTTCCCAATATCATGATGCCACCAGGAATCACCGAACAAACCACTCTCATAGAGATGGTAGGACTTGCTG**

           ****************************************************************************************************

                  1010      1020      1030      1040      1050      1060      1070      1080      1090      1100

Nichols **CTTTGATTGCAGAAGGAACGCTCGGCAGCGCCATTCAAACCGTGCTAGCCGCTGGCGCGCTCGCGGCGCTTGTATCGCAACTTGTACCGAACATAGAGCA**

Mexico A   **CTTTGATTGCAGAAGGAACGCTCGGCAGCGCCATTCAAACCGTGCTAGCCGCTGGCGCGCTCGCGGCGCTTGTATCGCAACTTGTACCGAACATAGAGCA**

PT_SIF     **CTTTGATTGCAGAAGGAACGCTCGGCAGCGCCATTCAAACCGTGCTAGCCGCTGGCGCGCTCGCGGCGCTTGTATCGCAACTTGTACCGAACATAGAGCA**

Sea81-4    **CTTTGATTGCAGAAGGAACGCTCGGCAGCGCCATTCAAACCGTGCTAGCCGCTGGCGCGCTCGCGGCGCTTGTATCGCAACTTGTACCGAACATAGAGCA**

SS14       **CTTTGATTGCAGAAGGAACGCTCGGCAGCGCCATTCAAACCGTGCTAGCCGCTGGCGCGCTCGCGGCGCTTGTATCGCAACTTGTACCGAACATAGAGCA**

           ****************************************************************************************************

                  1110      1120      1130      1140      1150      1160      1170      1180      1190      1200

Nichols **AGGAGTACGTGATGTCTTCCGCTCTTCCGATCCAAGAGTTGTCACTGCTAAACTTCTCGCTTTCCTTGAGCGCGCACCTATGAACGCGCTCAACATAGAC**

Mexico A   **AGGAGTACGTGATGTCTTCCGCTCTTCCGATCCAAGAGTTGTCACTGCTAAACTTCTCGCTTTCCTTGAGCGCGCACCTATGAACGCGCTCAACATAGAC**

PT_SIF     **AGGAGTACGTGATGTCTTCCGCTCTTCCGATCCAAGAGTTGTCACTGCTAAACTTCTCGCTTTCCTTGAGCGCGCACCTATGAACGCGCTCAACATAGAC**

Sea81-4    **AGGAGTACGTGATGTCTTCCGCTCTTCCGATCCAAGAGTTGTCACTGCTAAACTTCTCGCTTTCCTTGAGCGCGCACCTATGAACGCGCTCAACATAGAC**

SS14       **AGGAGTACGTGATGTCTTCCGCTCTTCCGATCCAAGAGTTGTCACTGCTAAACTTCTCGCTTTCCTTGAGCGCGCACCTATGAACGCGCTCAACATAGAC**

           ****************************************************************************************************

                  1210      1220      1230      1240      1250      1260      1270      1280      1290      1300

Nichols **GCGCTCCTGCGTATGCAGTGGAAGTGGCTCTCTTCTGGCATATACTTTGCCACCGCAGGCACTAATATCTTTGGCAAACGCGTCTTTGCTACCACTCGTG**

Mexico A  **GCGCTCCTGCGTATGCAGTGGAAGTGGCTCTCTTCTGGCATATACTTTGCCACCGCAGGCACTAATATCTTTGGCAAACGCGTCTTTGCTACCACTCGTG**

PT_SIF     **GCGCTCCTGCGTATGCAGTGGAAGTGGCTCTCTTCTGGCATATACTTTGCCACCGCAGGCACTAATATCTTTGGCAAACGCGTCTTTGCTACCACTCGTG**

Sea81-4    **GCGCTCCTGCGTATGCAGTGGAAGTGGCTCTCTTCTGGCATATACTTTGCCACCGCAGGCACTAATATCTTTGGCAAACGCGTCTTTGCTACCACTCGTG**

SS14       **GCGCTCCTGCGTATGCAGTGGAAGTGGCTCTCTTCTGGCATATACTTTGCCACCGCAGGCACTAATATCTTTGGCAAACGCGTCTTTGCTACCACTCGTG**

           ****************************************************************************************************

                  1310      1320      1330      1340      1350      1360      1370      1380      1390      1400

Nichols **CGCACTACTTTGATTTTGCCGGATTCCTTAAGCTCGAAACCAAAAGCGGTGACCCCTACACCCACCTGCTCACCGGCCTGAACGCCGGCGTCGAAGCACG**

Mexico A   **CGCACTACTTTGATTTTGCCGGATTCCTTAAGCTCGAAACCAAAAGCGGTGACCCCTACACCCACCTGCTCACCGGCCTGAACGCCGGCGTCGAAGCACG**

PT_SIF     **CGCACTACTTTGATTTTGCCGGATTCCTTAAGCTCGAAACCAAAAGCGGTGACCCCTACACCCACCTGCTCACCGGCCTGAACGCCGGCGTCGAAGCACG**

Sea81-4    **CGCACTACTTTGATTTTGCCGGATTCCTTAAGCTCGAAACCAAAAGCGGTGACCCCTACACCCACCTGCTCACCGGCCTGAACGCCGGCGTCGAAGCACG**

SS14       **CGCACTACTTTGATTTTGCCGGATTCCTTAAGCTCGAAACCAAAAGCGGTGACCCCTACACCCACCTGCTCACCGGCCTGAACGCCGGCGTCGAAGCACG**

           ****************************************************************************************************

Region I

                  1410      1420      1430      1440      1450      1460      1470      1480      1490      1500

Nichols **CGTGTACATCCCCCTCACCTACATCCGTTACAGAAATAACGGAGGGTACGAACTGAATGGAGCTGTGCCCCCTGGGACTATCAATATGCCAATTTTGGGG**

Mexico A   **CGTGTACATCCCCCTCACCTACGTCTTTTACAGAAATAACGGAGGGTACGAACTGAATAGAGTTGTGCCCTCTGGGATTATCAATATGCCAATTTTGGGG**

PT_SIF     **CGTGTACATCCCCCTCACCTACGTCTTTTACAGAAATAACGGAGGGTACGAACTGAATAGAGTTGTGCCCTCTGGGATTATCAATATGCCAATTTTGGGG**

Sea81-4    **CGTGTACATCCCCCTCACCTACGTCTTTTACAGAAATAACGGAGGGTACGAACTGAATAGAGTTGTGCCCTCTGGGATTATCAATATGCCAATTTTGGGG**

SS14       **CGTGTACATCCCCCTCACCTACGTCTTTTACAGAAATAACGGAGGGTACGAACTGAATAGAGTTGTGCCCTCTGGGATTATCAATATGCCAATTTTGGGG**

           ********************** **  ******************************* *** ******* ****** **********************

Region II

                  1510      1520      1530      1540      1550      1560      1570      1580      1590      1600

Nichols **AAGGCGTGGTGCAGCTATCGCATCCCCCTCGGTTCCCACGCCTGGCTTGCACCACACACATCCGTGCTCGGCACAACCAATCGCTTTAACATTATTAACC**

Mexico A   **AAGGCGTGGTGCAGCTATCGCATCCCCCTCGGTTCCCACGCCTGGCTTGCACCACACACATCCGTGCTCGGCACAACCAATCGCTTTAACATTATTAACG**

PT_SIF     **AAGGCGTGGTGCAGCTATCGCATCCCCCTCGGTTCCCACGCCTGGCTTGCACCACACACATCCGTGCTCGGCACAACCAATCGCTTTAACATTATTAACG**

Sea81-4    **AAGGCGTGGTGCAGCTATCGCATCCCCCTCGGTTCCCACGCCTGGCTTGCACCACACACATCCGTGCTCGGCACAACCAATCGCTTTAACATTATTAACG**

SS14       **AAGGCGTGGTGCAGCTATCGCATCCCCCTCGGTTCCCACGCCTGGCTTGCACCACACACATCCGTGCTCGGCACAACCAATCGCTTTAACATTATTAACC**

           ***************************************************************************************************

                  1610      1620      1630      1640      1650      1660      1670      1680      1690      1700

Nichols **CCGCGGGCAACCTGTTGAATGAACGAGCGCTCCAGTACCAGGTGGGACTGACGTTCAGTCCCTTCGAGAAGGTGGAGCTCAGCGCCCAGTGGGAACAGGG**

Mexico A   **CCGCGGGCAACCTGTTGAATGAACGAGCGCTCCAGTACCAGGTGGGACTGACGTTCAGTCCCTTCGAGAAGGTGGAGCTCAGCGCCCAGTGGGAACAGGG**

PT_SIF     **CCGCGGGCAACCTGTTGAATGAACGAGCGCTCCAGTACCAGGTGGGACTGACGTTCAGTCCCTTCGAGAAGGTGGAGCTCAGCGCCCAGTGGGAACAGGG**

Sea81-4    **CCGCGGGCAACCTGTTGAATGAACGAGCGCTCCAGTACCAGGTGGGACTGACGTTCAGTCCCTTCGAGAAGGTGGAGCTCAGCGCCCAGTGGGAACAGGG**

SS14       **CCGCGGGCAACCTGTTGAATGAACGAGCGCTCCAGTACCAGGTGGGACTGACGTTCAGTCCCTTCGAGAAGGTGGAGCTCAGCGCCCAGTGGGAACAGGG**

           ****************************************************************************************************

Region III

Region IV

                1710      1720      1730      1740      1750      1760      1770      1780      1790

Nichols **CGTGCTTGCTGACGCTCCTTACATGGGCATTGCCGAGAGCATCTGGTCCGAACGCCACTTCGGCACCCTTGTCTGCGGAATGAAAGTGACATGGTAA**

Mexico A   **CGTGCTTGCTGACGCTCCTTACATGGGTATTACTCAGAGCATCGGGTCCGACCGCCACTTCGGCACCCTTGTCTGTGGAATGAAAGTGACATGGTAA**

PT_SIF     **CGTGCTTGCTGACGCTCCTTACATGGGTATTACTCAGAGCATCGGGTCCGACCGCCACTTCGGCACCCTTGTCTGTGGAATGAAAGTGACATGGTAA**

Sea81-4    **GGTGCTCTCCGATGTTCCCTACATGGGCATTGCCGAGAGCATCTGGTCCGAACGCCACTTCGGCACCCTTGTCTGCGGAATGAAAGTGACATGGTAA**

SS14       **CGTGCTTGCTGACGCTCCTTACATGGGTATTACTCAGAGCATCGGGTCCGACCGCCACTTCGGCACCCTTGTCTGTGGAATGAAAGTGACATGGTAA**

           *****  * ** * *** ******** *** *  ******** ******* *********************** *********************
