# Supplementary material for: Sequence Variation of Rare Outer Membrane Protein β-Barrel Domains in Clinical Strains Provides Insights into the Evolution of Treponema pallidum subsp. pallidum, the Syphilis Spirochete
Source: mBio. 2018 Jun 12;9(3):e01006-18. doi: 10.1128/mBio.01006-18 (PMC6016234; doi:10.1128/mBio.01006-18)
Supplement: TABLE S2 [file mbo003183920st2.docx]

**Table S2. Distribution of clades and OMP allelic variants in *TPA* reference genomes**

| **Strain** | **Reference**  **Genome ID** | ***tp0548* type** | **Clade^1^** | ***tprC*** | ***tprD*** | ***bamA*** |
| --- | --- | --- | --- | --- | --- | --- |
| **CDC A** | GCF_001655275.1 | a | Nichols | Nichols | D | Nichols |
| **Chicago^2^** | GCF_000024485.1 | a | Nichols | Nichols | D | Nichols |
| **DAL-1** | GCF_000246815.1 | a | Nichols | Nichols | D | Nichols |
| **Nichols^3^** | GCF_000410535.2 | a | Nichols | Nichols | D | Nichols |
| **Sea81-4** | GCF_000604125.1 | b | Nichols | Sea81-4 | D2^6^ | Sea81-4 |
| **UW189B** | GCF_001655435.1 | d | Nichols | Sea81-4 | D2 | Sea81-4 |
| **SS14** | GCF_000410555.1 | f | SS14 | SS14 | D2 | SS14 |
| **PT_SIF0697^4^** | GCF_001712895.1 | f | SS14 | PT_SIF | D2 | SS14 |
| **PT_SIF0751^5^** | GCF_001761515.1 | g | SS14 | PT_SIF | D2 | SS14 |
| **UW074B** | GCF_001655395.1 | g | SS14 | PT_SIF | D2 | SS14 |
| **UW228B** | GCF_001655475.1 | f | SS14 | PT_SIF | D2 | SS14 |
| **UW254B** | GCF_001655515.1 | f | SS14 | PT_SIF | D2 | SS14 |
| **UW391B** | GCF_001655555.1 | f | SS14 | PT_SIF | D2 | SS14 |
| **Amoy** | GCF_001628695.1 | f | SS14 | Mexico A^6^ | D2^6^ | SS14 |
| **Mexico A** | GCF_000304295.1 | e | SS14 | Mexico A | D2 | Mexico A |

^1^ Clade designations based on both *tp0548* and *tp0558* sequences.

^2^ Chicago is representative of Chicago and Chicago Population at *tprC*, *tprD* and *bamA* loci.

^3^ Nichols is representative of Nichols, Nichols-Houston, and Nichols-Seattle at *tprC*, *tprD* and *bamA* loci.

^4^ PT_SIF0697 is representative of PT_SIF0697, PT_SIF0857, PT_SIF0908, PT_SIF1002, PT_SIF1020, PT_SIF1063, PT_SIF1135, PT_SIF1156, PT_SIF1167, PT_SIF1183, PT_SIF1196, and PT_SIF1242.

^5^ PT_SIF0751 is representative of PT_SIF0751, PT_SIF0877_3, PT_SIF0954, PT_SIF1127, PT_SIF1140, PT_SIF1142, PT_SIF1200, PT_SIF1252, PT_SIF1261, PT_SIF1278, PT_SIF1280, PT_SIF1299, and PT_SIF1348.

^6^ Partial sequence.
