# Supplementary material for: Sequence Variation of Rare Outer Membrane Protein β-Barrel Domains in Clinical Strains Provides Insights into the Evolution of Treponema pallidum subsp. pallidum, the Syphilis Spirochete
Source: mBio. 2018 Jun 12;9(3):e01006-18. doi: 10.1128/mBio.01006-18 (PMC6016234; doi:10.1128/mBio.01006-18)
Supplement: FIG S3 [file mbo003183920sf3.docx]

               10        20        30        40        50        60        70        80        90       100       110       120       130       140       150       160

*tprD*   GTGGGCAGGCAGGTGATGCAAGCGGGGGTACTTGCGGGCATGGTATGTGCTGCTTCTGGTTATGCAGGCGTACTCACTCCGCAGGTCAGTGGCACAGCCCAGCTCCAGTGGGGCATTGCGTTCCAGAAGAATCCACGCACTGGCCCGGGCAAGCACACCC

*tprD2*  GTGGGCAGGCAGGTGATGCAAGCGGGGGTACTTGCGGGCATGGTATGTGCTGCTTCTGGTTATGCAGGCGTACTCACTCCGCAGGTCAGTGGCACAGCCCAGCTCCAGTGGGGCATTGCGTTCCAGAAGAATCCACGCACTGGCCCGGGCAAGCACACCC

       ****************************************************************************************************************************************************************

              170       180       190       200       210       220       230       240       250       260       270       280       290       300       310       320

*tprD*   ATGGGTTTCGCACTACCAATAGTCTGACTATTTCCCTGCCGTTGGTGTCAAAGCACACCCACACCCGCCGAGGGGAGGCACGCTCAGGGGTGTGGGCACAGCTGCAGCTGAAGGACCTGGCAGTAGAGCTTGCGTCTTCTAAAAGCTCAACGGCCCTGTC

*tprD2*  ATGGGTTTCGCACTACCAATAGTCTGACTATTTCCCTGCCGTTGGTGTCAAAGCACACCCACACCCGCCGAGGGGAGGCACGCTCAGGGGTGTGGGCACAGCTGCAGCTGAAGGACCTGGCAGTAGAGCTTGCGTCTTCTAAAAGCTCAACGGCCCTGTC

       ****************************************************************************************************************************************************************

              330       340       350       360       370       380       390       400       410       420       430       440       450       460       470       480

*tprD*   CTTTACCAAACCTACCGCTTCCTTCCAGGCAACCCTGCACTGTTATGGGGCCTACCTGACAGTGGGTACCAGTCCTTCCTGTGTGGTTAACTTTGCCCAGCTGTGGAAACCCTTTGTCACCCGTGCCTATTCAGAAAAGGACACTCGCTATGCCCCTGGT

*tprD2*  CTTTACCAAACCTACCGCTTCCTTCCAGGCAACCCTGCACTGTTATGGGGCCTACCTGACAGTGGGTACCAGTCCTTCCTGTGTGGTTAACTTTGCCCAGCTGTGGAAACCCTTTGTCACCCGTGCCTATTCAGAAAAGGACACTCGCTATGCCCCTGGT

       ****************************************************************************************************************************************************************

              490       500       510       520       530       540       550       560       570       580       590       600       610       620       630       640

*tprD*   TTCTCCGGCTCCGGGGCAAAACTCGGCTACCAGGCCCACAATGTGGGAAACAGCGGAGTAGATGTGGACATCGGTTTCCTCTCCTTCCTTTCCAATGGTGCCTGGGATAGTACTGACACCACGCACAGCAAGTATGGCTTCGGGGCCGATGCAACGCTTT

*tprD2*  TTCTCCGGCTCCGGGGCAAAACTCGGCTACCAGGCCCACAATGTGGGAAACAGCGGAGTAGATGTGGACATCGGTTTCCTCTCCTTCCTTTCCAATGGTGCCTGGGATAGTACTGACACCACGCACAGCAAGTATGGCTTCGGGGCCGATGCAACGCTTT

       ****************************************************************************************************************************************************************

              650       660       670       680       690       700       710       720       730       740       750       760       770       780       790       800

*tprD*   CCTATGGCGTCGACCGTCAGCGGCTGCTTACGTTGGAGCTGGCAGGGAATGCCACACTGGACCAGAACTACGTTAAGGGTACCGAAGACTCCAAGAACGAAAACAAAACAGCACTCCTGTGGGGAGTAGGAGGCCGACTCACCCTCGAACCAGGCGCCGG

*tprD2*  CCTATGGCGTCGACCGTCAGCGGCTGCTTACGTTGGAGCTGGCAGGGAATGCCACACTGGACCAGAACTACGTTAAGGGTACCGAAGACTCCAAGAACGAAAACAAAACAGCACTCCTGTGGGGAGTAGGAGGCCGACTCACCCTCGAACCAGGCGCCGG

       ****************************************************************************************************************************************************************

              810       820       830       840       850       860       870       880       890       900       910       920       930       940       950       960

*tprD*   CTTCCGCTTCTCCTTCGCCCTCGACGCCGGTAACCAACACCAGAGTAACGCACATGCTCAGACCCAAGAGAGAGCTATCCTCAAAGCAAGGGAAGTGTTTAGACGGGTGGAGGGGAAACTCGTGCAGAACCTTCC-CAATATCATGATGCCACCA-----

*tprD2*  CTTCCGCTTCTCCTTCGCCCTCGACGCCGGTAACCAACACCAGAGTAACGCACAATTCTACGCTAGAATGGCTCCCTCACAGAGGGTCCATGAAGTCATCA--CTAGTCTTGGGGACACGC-TGCTGACCTCCCCGCAACAAGATGTTGTTTCATTCTTT

       ******************************************************     *  *   *  *    *    *  *  *     *****  * *  *  **   ***** ** * *** ** *   ** *** *  *** **   *

              970       980       990      1000      1010      1020      1030      1040      1050      1060      1070      1080      1090      1100      1110      1120

*tprD*   --GGAATCACCGAACAAAC-CACTCTCATAGAGATGGTAGGACTTGCTGCTTTGATTGCAGAAGGAACGCTCGGCAGCGCCAT-TCAAACCGTGCTAGCCGCTGGCGCGCTCGCGGCGCTTGTATCGCAACTTGTACCGAACATAGAGCAAGGAGTACGT

*tprD2*  GTGCAAGAACTGAGCAAAGGCAGTCTTCTGGAGAAAGCTGGCTTAGTAACGCTCTTGGCGCAGCGCACCATCGTCGGCTTAGCGTCAAGCGGTGGTTACCTAAGACATCTGAATGGCAAAGGCCTAGAAATAAACATGAGGCTCATAG--AGCAGCAGAA

Region I

         * **  ** ** ****  ** ***  * ****  *  **  * *   *  *  * **  *  * **  *** * **      **** * *** *  **   * *        ***    *  * * **     *     *  * **  ** ** *

             1130      1140      1150      1160      1170      1180      1190      1200      1210      1220      1230      1240      1250      1260      1270      1280

*tprD*   GATGTCTTCCGCTCTTCCGATCCAAGAGTTGTCACTGCTAAACTTCTCGCTTTCCTTGAGCGCGCACCTATGAACGCGCTCAACATAGACGCGCTCCTGCGTATGCAGTGGAAGTGGCTCTCTTCTGGCATATACTTTGCCACCGCAGGCACTAATATCT

*tprD*2  GAATCCTGACGCGCG---GATGCGGA---CAGCACT-CTTTATTTCCTGGTTGCAATTCACGTACACC----AAAACGCTCAACATAGACGCGCTCCTGCGTATGCAGTGGAGGTGGCTCTCTTCTGGCATATACTTTGCCACCGCAGGCACTAATATCT

Region II

       **   **  *** *    *** *         **** **  * ***  * ** *  *   **  ****    **  ************************************ ***********************************************

             1290      1300      1310      1320      1330      1340      1350      1360      1370      1380      1390      1400      1410      1420      1430      1440

*tprD*   TTGGCAAACGCGTCTTTGCTACCACTCGTGCGCACTACTTTGATTTTGCCGGATTCCTTAAGCTCGAAACCAAAAGCGGTGACCCCTACACCCACCTGCTCACCGGCCTGAACGCCGGCGTCGAAGCACGCGTGTACATCCCCCTCACCTACATCCGTTA

*tprD2*  TTGGAGAACGTGTTTTCTTTAAGAATCAAGCAGATCACTTTGATTTTGCCGGATTCCTCAAACTCGAAACCAAAAGCGGTGACCCCTACACCCACCTGCTCACCGGCCTGAACGCCGGCGTCGAAGCACGCGTGTACATCCCCCTCACCTACATCTTTTA

       ****  **** ** **   **  * **  **  *  ********************** ** *********************************************************************************************  ***

Region IV

Region III

             1450      1460      1470      1480      1490      1500      1510      1520      1530      1540      1550      1560      1570      1580      1590      1600

*tprD*   CAGAAATAACGGAGGGTACGAACTG-AATGGAGCTGTGCCCCCTGGGACTATCAATATGCCAATTTTGGGGAAGGCGTGGTGCAGCTATCGCATCCCCCTCGGTTCCCACGCCTGGCTTGCACCACACACATCCGTGCTCGGCACAACCAATCGCTTTAA

*tprD2*  CATAAATAACGGAGG-TGCGCAGTACAAGGGAAGTAATTCGGACGGCGTCATCAACACGCCTATCTTGAGCAAAGCGTGGTGCAGCTATCGCATCCCCCTCGGTTCCCACGCCTGGCTTGCACCACACACATCCGTGCTATGGGCAACAAACCGCTTCAA

       ** ************ * ** * *  ** ***  *    *    **    ***** * *** ** *** * ** *****************************************************************  *  **** ** ***** **

             1610      1620      1630      1640      1650      1660      1670      1680      1690      1700      1710      1720      1730      1740      1750      1760

*tprD*   CATTATTAACCCCGCGGGCAACCTGTTGAATGAACGAGCGCTCCAGTACCAGGTGGGACTGACGTTCAGTCCCTTCGAGAAGGTGGAGCTCAGCGCCCAGTGGGAACAGGGCGTGCTTGCTGACGCTCCTTACATGGGCATTGCCGAGAGCATCTGGTCC

*tprD*2  CCACAACCAGAGCGGGGATGCGCTCCTGCGTGAGCACGCGCTCCAGTACCAGGTGGGACTGACGTTCAGTCCCTTCGAGAAGGTGGAGCTCAGCGCCCAGTGGGAACAGGGCGTGCTTGCTGACGCTCCTTACATGGGCATTGCCGAGAGCATCTGGTCC

       *   *   *   ** **     **  **  *** *  ***************************************************************************************************************************

             1770      1780      1790      1800

*tprD*   GAACGCCACTTCGGCACCCTTGTCTGCGGAATGAAAGTGACATGG

*tprD2*  GAACGCCACTTCGGCACCCTTGTCTGCGGAATGAAAGTGACATGG

       *********************************************
