# Supplementary material for: Sequence Variation of Rare Outer Membrane Protein β-Barrel Domains in Clinical Strains Provides Insights into the Evolution of Treponema pallidum subsp. pallidum, the Syphilis Spirochete
Source: mBio. 2018 Jun 12;9(3):e01006-18. doi: 10.1128/mBio.01006-18 (PMC6016234; doi:10.1128/mBio.01006-18)
Supplement: FIG S4 [file mbo003183920sf4.docx]

                  10        20        30        40        50        60        70        80        90       100

Nichols   ATGCTCAAAAAAGCCAGTGCCTTCCTAATTGCAAGTTGTTGTGTGATGTCGCTGGCGTGGGCACAGGCAAACGACAATTGGTACGAGGGAAAGCCTATCT

Mexico A  ATGCTCAAAAAAGCCAGTGCCTTCCTAATTGCAAGTTGTTGTGTGATGTCGCTGGCGTGGGCACAGGCAAACGACAATTGGTACGAGGGAAAGCCTATCT

Sea81-4   ATGCTCAAAAAAGCCAGTGCCTTCCTAATTGCAAGTTGTTGTGTGATGTCGCTGGCGTGGGCACAGGCAAACGACAATTGGTACGAGGGAAAGCCTATCT

SS14      ATGCTCAAAAAAGCCAGTGCCTTCCTAATTGCAAGTTGTTGTGTGATGTCGCTGGCGTGGGCACAGGCAAACGACAATTGGTACGAGGGAAAGCCTATCT

          ****************************************************************************************************

                 110       120       130       140       150       160       170       180       190       200

Nichols   CTGCGATTAGTTTTGAGGGGCTCGAATATATTGCTCGCGGCCAGTTGGACACGATTTTTTCTCAATACAAGGGACAAAAGTGGACCTATGAGCTGTACCT

Mexico A  CTGCGATTAGTTTTGAGGGGCTCGAATATATTGCTCGCGGCCAGTTGGACACGATTTTTTCTCAATACAAGGGACAAAAGTGGACCTATGAGCTGTACCT

Sea81-4   CTGCGATTAGTTTTGAGGGGCTCGAATATATTGCTCGCGGCCAGTTGGACACGATTTTTTCTCAATACAAGGGACAAAAGTGGACCTATGAGCTGTACCT

SS14      CTGCGATTAGTTTTGAGGGGCTCGAATATATTGCTCGCGGCCAGTTGGACACGATTTTTTCTCAATACAAGGGACAAAAGTGGACCTATGAGCTGTACCT

          ****************************************************************************************************

                 210       220       230       240       250       260       270       280       290       300

Nichols   GGAGATACTGCAAAAGGTCTATGACCTTGAGTACTTTTCTGAAGTTTCGCCTAAGGCGGTGCCCACCGATCCGGAGTATCAGTATGTGATGCTACAGTTC

Mexico A  GGAGATACTGCAAAAGGTCTATGACCTTGAGTACTTTTCTGAAGTTTCGCCTAAGGCGGTGCCCACCGATCCGGAGTATCAGTATGTGATGCTACAGTTC

Sea81-4   GGAGATACTGCAAAAGGTCTATGACCTTGAGTACTTTTCTGAAGTTTCGCCTAAGGCGGTGCCCACCGATCCGGAGTATCAGTATGTGATGCTACAGTTC

SS14      GGAGATACTGCAAAAGGTCTATGACCTTGAGTACTTTTCTGAAGTTTCGCCTAAGGCGGTGCCCACCGATCCGGAGTATCAGTATGTGATGCTACAGTTC

          ****************************************************************************************************

                 310       320       330       340       350       360       370       380       390       400

Nichols   ACGGTAAAGGAGCGTCCTTCGGTGAAGGGCATCAAGATGGTAGGGAACAGCCAAATCCGCAGTGGGGACCTTTTGTCTAAAATCCTCCTGAAAAAGGGAG

Mexico A  ACGGTAAAGGAGCGTCCTTCGGTGAAGGGCATCAAGATGGTAGGGAACAGCCAAATCCGCAGTGGGGACCTTTTGTCTAAAATCCTCCTGAAAAAGGGAG

Sea81-4   ACGGTAAAGGAGCGTCCTTCGGTGAAGGGCATCAAGATGGTAGGGAACAGCCAAATCCGCAGTGGGGACCTTTTGTCTAAAATCCTCCTGAAAAAGGGAG

SS14      ACGGTAAAGGAGCGTCCTTCGGTGAAGGGCATCAAGATGGTAGGGAACAGCCAAATCCGCAGTGGGGACCTTTTGTCTAAAATCCTCCTGAAAAAGGGAG

          ****************************************************************************************************

                 410       420       430       440       450       460       470       480       490       500

Nichols   ACATTTACAATGAAGTAAAGATGAAGGTGGACCAAGAGTCGCTCAGGCGTCATTACCTGGACCAGGGCTATGCGGCGGTTAAGATATCCTGCGAGGCAAA

Mexico A  ACATTTACAATGAAGTAAAGATGAAGGTGGACCAAGAGTCGCTCAGGCGTCATTACCTGGACCAGGGCTATGCGGCGGTTAAGATATCCTGCGAGGCAAA

Sea81-4   ACATTTACAATGAAGTAAAGATGAAGGTGGACCAAGAGTCGCTCAGGCGTCATTACCTGGACCAGGGCTATGCGGCGGTTAAGATATCCTGCGAGGCAAA

SS14      ACATTTACAATGAAGTAAAGATGAAGGTGGACCAAGAGTCGCTCAGGCGTCATTACCTGGACCAGGGCTATGCGGCGGTTAAGATATCCTGCGAGGCAAA

          ****************************************************************************************************

                 510       520       530       540       550       560       570       580       590       600

Nichols   AACTGAGGCGGGGGGCGTGGTGGTACAGTTTACCATCCAGGAAGGTAAGCAGACTGTTGTCTCGCGGATACAGTTTAAGGGAAATAAGGCGTTTACCGAG

Mexico A  AACTGAGGCGGGGGGCGTGGTGGTACAGTTTACCATCCAGGAAGGTAAGCAGACTGTTGTCTCGCGGATACAGTTTAAGGGAAATAAGGCGTTTACCGAG

Sea81-4   AACTGAGGCGGGGGGCGTGGTGGTACAGTTTACCATCCAGGAAGGTAAGCAGACTGTTGTCTCGCGGATACAGTTTAAGGGAAATAAGGCGTTTACCGAG

SS14      AACTGAGGCGGGGGGCGTGGTGGTACAGTTTACCATCCAGGAAGGTAAGCAGACTGTTGTCTCGCGGATACAGTTTAAGGGAAATAAGGCGTTTACCGAG

          ****************************************************************************************************

                 610       620       630       640       650       660       670       680       690       700

Nichols   TCGGTGCTCAAGAAGGTGCTTTCCACGCAGGAGGCGCGTTTTTTGACCAGTGGGGTGTTCAAGGAGAATGCGCTGGAAGCGGATAAGGCGGCAGTCCACT

Mexico A  TCGGTGCTCAAGAAGGTGCTTTCCACGCAGGAGGCGCGTTTTTTGACCAGTGGGGTGTTCAAGGAGAATGCGCTGGAAGCGGATAAGGCGGCAGTCCACT

Sea81-4   TCGGTGCTCAAGAAGGTGCTTTCCACGCAGGAGGCGCGTTTTTTGACCAGTGGGGTGTTCAAGGAGAATGCGCTGGAAGCGGATAAGGCGGCAGTCCACT

SS14      TCGGTGCTCAAGAAGGTGCTTTCCACGCAGGAGGCGCGTTTTTTGACCAGTGGGGTGTTCAAGGAGAATGCGCTGGAAGCGGATAAGGCGGCAGTCCACT

          ****************************************************************************************************

                 710       720       730       740       750       760       770       780       790       800

Nichols   CATACTATGCAGAGAGGGGATACATTGACGCGCGGGTAGAAGGCGTGGCAAAGACGGTTGATAAAAAAACTGACGCCAGTCGCAATCTGGTTACGCTTAC

Mexico A  CATACTATGCAGAGAGGGGATACATTGACGCGCGGGTAGAAGGCGTGGCAAAGACGGTTGATAAAAAAACTGACGCCAGTCGCAATCTGGTTACGCTTAC

Sea81-4   CATACTATGCAGAGAGGGGATACATTGACGCGCGGGTAGAAGGCGTGGCAAAGACGGTTGATAAAAAAACTGACGCCAGTCGCAATCTGGTTACGCTTAC

SS14      CATACTATGCAGAGAGGGGATACATTGACGCGCGGGTAGAAGGCGTGGCAAAGACGGTTGATAAAAAAACTGACGCCAGTCGCAATCTGGTTACGCTTAC

          ****************************************************************************************************

                 810       820       830       840       850       860       870       880       890       900

Nichols   GTACACTGTGGTGGAAGGTGAGCAGTACCGCTACGGCGGGGTTACCATTGTGGGTAACCAGATTTTTAGCACCGAGGAGCTGCAGGCAAAAATTAGGCTC

Mexico A  GTACACTGTGGTGGAAGGTGAGCAGTACCGCTACGGCGGGGTTACCATTGTGGGTAACCAGATTTTTAGCACCGAGGAGCTGCAGGCAAAAATTAGGCTC

Sea81-4   GTACACTGTGGTGGAAGGTGAGCAGTACCGCTACGGCGGGGTTACCATTGTGGGTAACCAGATTTTTAGCACCGAGGAGCTGCAGGCAAAAATTAGGCTC

SS14      GTACACTGTGGTGGAAGGTGAGCAGTACCGCTACGGCGGGGTTACCATTGTGGGTAACCAGATTTTTAGCACCGAGGAGCTGCAGGCAAAAATTAGGCTC

          ****************************************************************************************************

                 910       920       930       940       950       960       970       980       990      1000

Nichols   AAGCGCGGGGCCATCATGAATATGGTGGCCTTTGAGCAGGGCTTTCAGGCGCTGGCGGATGCGTATTTTGAAAACGGATACACGTCAAATTACCTGAACA

Mexico A  AAGCGCGGGGCCATCATGAATATGGTGGCCTTTGAGCAGGGCTTTCAGGCGCTGGCGGATGCGTATTTTGAAAACGGATACACGTCAAATTACCTGAACA

Sea81-4   AAGCGCGGGGCCATCATGAATATGGTGGCCTTTGAGCAGGGCTTTCAGGCGCTGGCGGATGCGTATTTTGAAAACGGATACACGTCAAATTACCTGAACA

SS14      AAGCGCGGGGCCATCATGAATATGGTGGCCTTTGAGCAGGGCTTTCAGGCGCTGGCGGATGCGTATTTTGAAAACGGATACACGTCAAATTACCTGAACA

          ****************************************************************************************************

                1010      1020      1030      1040      1050      1060      1070      1080      1090      1100

Nichols   AAGAAGAACACCGGGACACGGCGGAGAAAACGCTTTCGTTTAAGATCACGGTGGTGGAGCGCGAGCGCAGCCACGTCGAGCACATTATCATTAAGGGAAC

Mexico A  AAGAAGAACACCGGGACACGGCGGAGAAAACGCTTTCGTTTAAGATCACGGTGGTGGAGCGCGAGCGCAGCCACGTCGAGCACATTATCATTAAGGGAAC

Sea81-4   AAGAAGAACACCGGGACACGGCGGAGAAAACGCTTTCGTTTAAGATCACGGTGGTGGAGCGCGAGCGCAGCCACGTCGAGCACATTATCATTAAGGGAAC

SS14      AAGAAGAACACCGGGACACGGCGGAGAAAACGCTTTCGTTTAAGATCACGGTGGTGGAGCGCGAGCGCAGCCACGTCGAGCACATTATCATTAAGGGAAC

          ****************************************************************************************************

                1110      1120      1130      1140      1150      1160      1170      1180      1190      1200

Nichols   GAAGAATACAAAAGACGAGGTTATCCTGCGTGAAATGCTGCTGAAACCGGGGGATGTGTTCTCTAAGTCAAAGTTTACGGATAGCTTGCGCAATCTGTTC

Mexico A  GAAGAATACAAAAGACGAGGTTATCCTGCGTGAAATGCTGCTGAAACCGGGGGATGTGTTCTCTAAGTCAAAGTTTACGGATAGCTTGCGCAATCTGTTC

Sea81-4   GAAGAATACAAAAGACGAGGTTATCCTGCGTGAAATGCTGCTGAAACCGGGGGATGTGTTCTCTAAGTCAAAGTTTACGGATAGCTTGCGCAATCTGTTC

SS14      GAAGAATACAAAAGACGAGGTTATCCTGCGTGAAATGCTGCTGAAACCGGGGGATGTGTTCTCTAAGTCAAAGTTTACGGATAGCTTGCGCAATCTGTTC

          ****************************************************************************************************

                1210      1220      1230      1240      1250      1260      1270      1280      1290      1300

Nichols   AACCTGCGCTATTTCTCGTCGCTGGTGCCGGATGTGCGGCCCGGCTCTGAGCAGGACCTGGTGGACATTATCCTGAATGTGGAGGAGCAGTCGACGGCAA

Mexico A  AACCTGCGCTATTTCTCGTCGCTGGTGCCGGATGTGCGGCCCGGCTCTGAGCAGGACCTGGTGGACATTATCCTGAATGTGGAGGAGCAGTCGACGGCAA

Sea81-4   AACCTGCGCTATTTCTCGTCGCTGGTGCCGGATGTGCGGCCCGGCTCTGAGCAGGACCTGGTGGACATTATCCTGAATGTGGAGGAGCAGTCGACGGCAA

SS14      AACCTGCGCTATTTCTCGTCGCTGGTGCCGGATGTGCGGCCCGGCTCTGAGCAGGACCTGGTGGACATTATCCTGAATGTGGAGGAGCAGTCGACGGCAA

          ****************************************************************************************************

                1310      1320      1330      1340      1350      1360      1370      1380      1390      1400

Nichols   ACGTGCAGTTTGGGGTGACGTTTTCTGGGGTGGGGGAGGCAGGCACGTTCCCGCTTTCGCTCTTTTGTCAGTGGGAAGAAAAGAATTTTTTGGGAAAAGG

Mexico A  ACGTGCAGTTTGGGGTGACGTTTTCTGGGGTGGGGGAGGCAGGCACGTTCCCGCTTTCGCTCTTTTGTCAGTGGGAAGAAAAGAATTTTTTGGGAAAAGG

Sea81-4   ACGTGCAGTTTGGGGTGACGTTTTCTGGGGTGGGGGAGGCAGGCACGTTCCCGCTTTCGCTCTTTTGTCAGTGGGAAGAAAAGAATTTTTTGGGAAAAGG

SS14      ACGTGCAGTTTGGGGTGACGTTTTCTGGGGTGGGGGAGGCAGGCACGTTCCCGCTTTCGCTCTTTTGTCAGTGGGAAGAAAAGAATTTTTTGGGAAAAGG

          ****************************************************************************************************

                1410      1420      1430      1440      1450      1460      1470      1480      1490      1500

Nichols   GAATGAAATTTCAGTAAATGCAACCTTGGGGTCTGAGGCGCAGAGCCTGAAGCTCGGGTATGTGGAGCGCTGGTTTCTGGGCTCTCCGCTGACGGTGGGC

Mexico A  GAATGAAATTTCAGTAAATGCAACCTTGGGGTCTGAGGCGCAGAGCCTGAAGCTCGGGTATGTGGAGCGCTGGTTTCTGGGCTCTCCGCTGACGGTGGGC

Sea81-4   GAATGAAATTTCAGTAAATGCAACCTTGGGGTCTGAGGCGCAGAGCCTGAAGCTCGGGTATGTGGAGCGCTGGTTTCTGGGCTCTCCGCTGACGGTGGGC

SS14      GAATGAAATTTCAGTAAATGCAACCTTGGGGTCTGAGGCGCAGAGCCTGAAGCTCGGGTATGTGGAGCGCTGGTTTCTGGGCTCTCCGCTGACGGTGGGC

          ****************************************************************************************************

Region I

                1510      1520      1530      1540      1550      1560      1570      1580      1590      1600

Nichols   TTTGACTTTGAACTTACGCACAAAAATCTCTTTGTGTACCGCGCGGGTTCATACGGCAACGGGCTGCCGCACCCGTACACGAGCAGGGAGCAGTGGGCTA

Mexico A  TTTGACTTTGAACTTACGCACAAAAATCTCTTTGTGTACCGCGCGGGTTCATACGGCAACGGGCTGCCGCACCCGTACACGAGCAGGGAGCAGTGGGCTA

Sea81-4   TTTGACTTTGAACTTACGCACAAAAATCTCTTTGTGTACCGCGCGGGTTCATACGGCAACGGGCTGCCGCACCCGTACACGAGCAGGGAGCAGTGGGCTA

SS14      TTTGACTTTGAACTTACGCACAAAAATCTCTTTGTGTACCGCGC**A**GGT**G**CA**A**A**A**GGCAACGGGCTGCCGCACCCGTAC**GT**GAGCA**A**GGAGCA**C**TGGGCTA

          ******************************************** *** ** * ************************  ***** ****** *******

                1610      1620      1630      1640      1650      1660      1670      1680      1690      1700

Nichols   GTTCCCCTGGGCTGGCAGAATCGTTTCGCCTCAAGTATTCGCGCTTTGAGTCCGCCATCGGCGCGCACACCGGGTACCAGTGGTATCCGCGCTATGCGGT

Mexico A  GTTCCCCTGGGCTGGCAGAATCGTTTCGCCTCAAGTATTCGCGCTTTGAGTCCGCCATCGGCGCGCACACCGGGTACCAGTGGTATCCGCGCTATGCGGT

Sea81-4   GTTCCCCTGGGCTGGCAGAATCGTTTCGCCTCAAGTATTCGCGCTTTGAGTCCGCCATCGGCGCGCACACCGGGTACCAGTGGTATCCGCGCTATGCGGT

SS14      ATTCCCCTGGGCTGGCAGAATCGTTTCGCCTCAAGTATTCGCGCTTTGAGTCCGCCATCGGCGCGCACACCGGGTACCAGTGGTATCCGCGCTATGCGGT

           ***************************************************************************************************

Region II

                1710      1720      1730      1740      1750      1760      1770      1780      1790      1800

Nichols   CATTAGGGTGAACGGGGGGGTGGACTTTCGGGTTGTAAAGAATTTTTACGATAAGGATAACAATCAGCCCTTCGACCTGACCGTAAAAGAGCAGCTGAAC

Mexico A  CATTAGGGTGAACGGGGGGGTGGACTTTCGGGTTGTAAAGAATTTTTACGATAAGGATAACAATCAGCCCTTCGACC**A**GACCGTAAAAGAGCAGCTGAAC

Sea81-4   CATTAGGGTGAACGGGGGGGTGGACTTTCGGGTTGTAAAGAATTTTTACGATAAGGATAACAATCAGCCCTTCGACCTGACCGTAAAAGAGCAGCTGAAC

SS14      CATTAGGGTGAACGGGGGGGTGGACTTTCGGGTTGTAAAGAATTTTTACGATAAGGATAACAATCAGCCCTTCGACCTGACCGTAAAAGAGCAGCTGAAC

          ***************************************************************************** **********************

                1810      1820      1830      1840      1850      1860      1870      1880      1890      1900

Nichols   TGGACCAGTATCAATTCGTTTTGGACGAGCGTTTCGTTTGACGGGCGTGACTTTGCGTACGACCCGTCCAGCGGCTGGTTTTTAGGACAGCGCTGTACGT

Mexico A  TGGACCAGTATCAATTCGTTTTGGACGAGCGTTTCGTTTGACGGGCGTGACTTTGCGTACGACCCGTCCAGCGGCTGGTTTTTAGGACAGCGCTGTACGT

Sea81-4   TGGACCAGTATCAATTCGTTTTGGACGAGCGTTTCGTTTGACGGGCGTGACTTTGCGTACGACCCGTCCAGCGGCTGGTTTTTAGGACAGCGCTGTACGT

SS14      TGGACCAGTATCAATTCGTTTTGGACGAGCGTTTCGTTTGACGGGCGTGACTTTGCGTACGACCCGTCCAGCGGCTGGTTTTTAGGACAGCGCTGTACGT

          ****************************************************************************************************

Region III

                1910      1920      1930      1940      1950      1960      1970      1980      1990      2000

Nichols   TCAACGGGCTCGTTCCCTTTCTCGAAAAAGAGCATTCGTTTCGCTCCGACACCAAGGCCGAGTTCTACGTTACCCTGCTCAATTATCCGGTCTCTGCCGT

Mexico A  TCAACGGGCTCGTTCCCT**G**TCTCGAAAAAGAGCATTCGTTTCGCTCCGACACCAAGGCCGAGTTCTACGTTACCCTGCTCAATTATCCGGTCTCTGCCGT

Sea81-4   TCAACGGGCTCGTTCCCTTTCTCGAAAAAGAGCATTCGTTTCGCTCCGACACCAAGGCCGAGTTCTACGTTACCCTGCTCAATTATCCGGTCTCTGCCGT

SS14      TCAACGGGCTCGTTCCCT**G**TCTCGAAAAAGAGCATTCGTTTCGCTCCGACACCAAGGCCGAGTTCTACGTTACCCTGCTCAATTATCCGGTCTCTGCCGT

          ****************** *********************************************************************************

                2010      2020      2030      2040      2050      2060      2070      2080      2090      2100

Nichols   GTGGAACTTAAAGTTTGTCTTGGCTTTCTACACCGGTGTGTCCGTTCAAACGTATTATGGACGGAGGAAAAGCGAAAACGGAAAGGGCAACGGGGTGCGG

Mexico A  GTGGAACTTAAAGTTTGTCTTGGCTTTCTACACCGGTGTGTCCGTTCAAACGTATTATGGACGGAGGAAAAGCGAAAACGGAAAGGGCAACGGGGTGCGG

Sea81-4   GTGGAACTTAAAGTTTGTCTTGGCTTTCTACACCGGTGTGTCCGTTCAAACGTATTATGGACGGAGGAAAAGCGAAAACGGAAAGGGCAACGGGGTGCGG

SS14      GTGGAACTTAAAGTTTGTCTTGGCTTTCTACACCGGTGTGTCCGTTCAAACGTATTATGGACGGAGGAAAAGCGAAAACGGAAAGGGCAACGGGGTGCGG

          ****************************************************************************************************

                2110      2120      2130      2140      2150      2160      2170      2180      2190      2200

Nichols   TCCGGCGCGCTGGTAATAGACGGCGTGCTGGTAGGGCGCGGGTGGAGCGAAGACGCAAAGAAAAACACCGGAGACCTGCTGCTCCACCACTGGATTGAGT

Mexico A  TCCGGCGCGCTGGTAATAGACGGCGTGCTGGTAGGGCGCGGGTGGAGCGAAGACGCAAAGAAAAACACCGGAGACCTGCTGCTCCACCACTGGATTGAGT

Sea81-4   TCCGGCGCGCTGGTAATAGACGGCGTGCTGGTAGGGCGCGGGTGGAGCGAAGACGCAAAGAAAAACACCGGAGACCTGCTGCTCCACCACTGGATTGAGT

SS14      TCCGGCGCGCTGGTAATAGACGGCGTGCTGGTAGGGCGCGGGTGGAGCGAAGACGCAAAGAAAAACACCGGAGACCTGCTGCTCCACCACTGGATTGAGT

          ****************************************************************************************************

                2210      2220      2230      2240      2250      2260      2270      2280      2290      2300

Nichols   TCCGCTGGCCGCTGGCGCACGGCATTGTGTCCTTTGACTTTTTCTTTGATGCGGCAATGGTGTACAACATCGAAAGTCAGTCCCCAAACGGGTCATCGTC

Mexico A  TCCGCTGGCCGCTGGCGCACGGCATTGTGTCCTTTGACTTTTTCTTTGATGCGGCAATGGTGTACAACATCGAAAGTCAGTCCCCAAACGGGTCATCGTC

Sea81-4   TCCGCTGGCCGCTGGCGCACGGCATTGTGTCCTTTGACTTTTTCTTTGATGCGGCAATGGTGTACAACATCGAAAGTCAGTCCCCAAACGGGTCATCGTC

SS14      TCCGCTGGCCGCTGGCGCACGGCATTGTGTCCTTTGACTTTTTCTTTGATGCGGCAATGGTGTACAACATCGAAAGTCAGTCCCCAAACGGGTCATCGTC

          ****************************************************************************************************

Region IV

                2310      2320      2330      2340      2350      2360      2370      2380      2390      2400

Nichols   CGCCAGCAGCTCCAGCAGCAGCAGTAGTAGTAGCAGTAGAACCACCAGCTCTGAAGGACTGTACAAAATGAGCTACGGTCCGGGGCTGCGCTTTACATTG

Mexico A  CGCCAGCAGCTCCAGCAGCAGCAG**C**AGTAGTAGCA**---------------**CTGAAGGACTGTACAAAATGAGCTACGGTCCGGGGCTGCGCTTTACATTG

Sea81-4   CGCCAGCAGCTCCAGCAGCAGCAGTAGTAGTAGCAGTAG**T**A**GT**A**G**CAGCTCTGAAGGACTGTACAAAATGAGCTACGGTCCGGGGCTGCGCTTTACATTG

SS14      CGCCAGCAGCTCCAGCAGCAGCAGTAGTAGTAGCAGTAG**C**ACCACCAGCTCTGAAGGACTGTACAAAATGAGCTACGGTCCGGGGCTGCGCTTTACATTG

          ************************ **********               **************************************************

Region VI

Region V

                2410      2420      2430      2440      2450      2460      2470      2480      2490      2500

Nichols   CCGCAATTTCCGTTAAAATTGGCGTTCGCAAACACCTTCACGTCACCCGGCGGCATCCCAAAAACAAAGAAAAATTGGAATTTTGTGTTGTCGTTCACGG

Mexico A  CCGCAATTTCCGTTAAAATTGGCGTTCGCAAACACCTTCACGTCA**G**CCGGCGGCATCCCAAAAACAAAGAAAAATTGGAATTTTGTGTTGTCGTTCACGG

Sea81-4   CCGCAATTTCCGTTAAAATTGGCGTTCGCAAACACCTTCACGTCACCCGGCGGCATCCCAAAAACAAAGAAAAATTGGAATTTTGTGTTGTCGTTCACGG

SS14      CCGCAATTTCCGTTAAAATTGGCGTTCGCAAACACCTTCACGTCACCCGGCGGCATCCCAAAAACAAAGAAA**G**ATTGGAATTTTGTGTTGTCGTTCACGG

          ********************************************* ************************** ***************************

                2510

Nichols   TAAATAATTTGTAG

Mexico A  TAAATAATTTGTAG

Sea81-4   TAAATAATTTGTAG

SS14      TAAATAATTTGTAG

          **************
