# Supplementary material for: Sequence Variation of Rare Outer Membrane Protein β-Barrel Domains in Clinical Strains Provides Insights into the Evolution of Treponema pallidum subsp. pallidum, the Syphilis Spirochete
Source: mBio. 2018 Jun 12;9(3):e01006-18. doi: 10.1128/mBio.01006-18 (PMC6016234; doi:10.1128/mBio.01006-18)
Supplement: TABLE S3 [file mbo003183920st3.docx]

**Table S3. Oligonucleotide primers used in these studies*.***

| **Gene** | **Primer Designation** | **Sequence (5**'**-3**'**)** | **Amplified region** | **Genome Coordinates** |
| --- | --- | --- | --- | --- |
| *tp0548* | tp0548^Flanking^-5’ | GCGTGGTGGTGAGTTCTTCT | *tp0548* variable region, 1^st^ PCR | 593206:593225 |
|  | tp0548^Flanking^-3’ | CGTTTCGGTGTGTGAGTCAT | *tp0548* variable region, 1^st^ PCR | 593628:593647 |
|  | tp0548 5’ | GGTCCCTATGATATCGTGTTCG | *tp0548* variable region, 2^nd^ PCR | 593287:593308 |
|  | tp0548 3’ | GTCATGGATCTGCGAGTGG | *tp0548* variable region, 2^nd^ PCR | 593614:593632 |
| *tp0558* | tp0558^Flanking^-5’ | GAAAGCACTCCCCAATACGA | *tp0558* variable region, 1^st^ PCR | 605642:605661 |
|  | tp0558^Flanking^-3’ | ACGTGAGGGTTTATGCTTCG | *tp0558* variable region, 1^st^ PCR | 606839:606858 |
|  | tp0558 5’ | CTCACTGCAGCGTTACTTGC | *tp0558* variable region, 2^nd^ PCR | 606094:606113 |
|  | tp0558 3’ | TCAACCGAGTGAAGCTACGA | *tp0558* variable region, 2^nd^ PCR | 606647:606666 |
| *tprC* | tprC^Flanking-^5' | TTCCTTGAGCGCGCACCT | TprC β-barrel,  1^st^ PCR | 135530:135547 |
|  | tprC^Flanking-^3' | CACCCCTGTTGCGCGCAGC | TprC β-barrel,  1^st^ PCR | 134702:134720 |
|  | tprC 5' | AACATAGACGCGCTCCTGC | TprC β-barrel,  2^nd^ PCR | 135499:135517 |
|  | tprC 3' | TTACCATGTCACTTTCATTCC | TprC β-barrel,  2^nd^ PCR | 134912:134932 |
| *tprD* | tprD^Flanking-^5' | GTAACCAACACCAGAGTAAC | TprD β-barrel and central, 1^st^ PCR | 153262:153281^2^ |
|  | tprD^Flanking-^3' | CCAGTTTCAGATGCAATGCCC | TprD β-barrel and central, 1^st^ PCR | 152038:152058^2^ |
|  | Mexico A tprD 5' ^1^ | GGGGACACGCTGCTGACC | TprD β-barrel and central, 2^nd^ PCR | 153235:153252^2^ |
|  | tprD 3' | TTACCATGTCACTTTCAT | TprD β-barrel and central, 2^nd^ PCR | 152371:152388^2^ |
| *bamA* | bamA^Flanking-^5' | CCCGGCTCTGAGCAGGACCTG | BamA β-barrel,  1^st^ PCR | 346722:346742 |
|  | bamA^Flanking-^3' | CTACAAATTATTTACCGTGAA | BamA β-barrel,  1^st^ PCR | 347976:347996 |
|  | bamA 5' | CTGAATGTGGAGGAGCAGTCG | BamA β-barrel,  2^nd^ PCR | 346755:346775 |
|  | bamA 3' | CGACAACACAAAATTCCAATT | BamA β-barrel,  2^nd^ PCR | 347955:347975 |
|  | SDTP0326L^3^ | CGTTTAAGATCACGGTGGTG | BamA β-barrel,  1^st^ PCR | 346519:346538 |
|  | tp0326 W-R^3^ | TGCAGCAGCGACGTAAGGAG | BamA β-barrel,  1^st^ PCR | 348083:348102 |
|  | tp0326-345595F^3^ | CCTGAATGTGGAGGAGCAGT | BamA β-barrel,  2^nd^ PCR | 346754:346773 |
|  | tp0326R_inner_R^3^ | AGCCCCGGACCGTTTCAAAACG | BamA β-barrel,  2^nd^ PCR | 348008:348029 |

^1^ Primer is specific for *tprD2* from Mexico A-like strains. Primer TprC 5’ was used as the 2^nd^ reaction forward primer to amplify *tprD* from Nichols-like strains.

^2^ Genome coordinates for *tprD2* correspond to the *TPA* Mexico A genome (GCF_000304295.1). All other coordinates correspond to the Nichols genome (GCF_000410535.2).

^3^ Primers used for amplification of the *bamA* sequence in clinical samples from Czech Republic.
