# Supplementary material for: Sequence Variation of Rare Outer Membrane Protein β-Barrel Domains in Clinical Strains Provides Insights into the Evolution of Treponema pallidum subsp. pallidum, the Syphilis Spirochete
Source: mBio. 2018 Jun 12;9(3):e01006-18. doi: 10.1128/mBio.01006-18 (PMC6016234; doi:10.1128/mBio.01006-18)
Supplement: TABLE S4 [file mbo003183920st4.docx]

**Table S4. Accession numbers of *tp0558 and* β-barrel-encoding sequences from clinical samples.**

| **Patient** | ***tp0558*** | ***tprC* β-barrel** | ***tprD* β-barrel** | ***bamA* β-barrel^1^** |
| --- | --- | --- | --- | --- |
| Cali_77 | ND^1^ | JQ418492 | ND | KP713715 |
| Cali_133 | KU341836 | KU171114 | KU201355 | KU201363 |
| Cali_101 | MF448242 | KU171110 | KU201351 | KU201359 |
| Cali_127 | MF448245 | KU171113 | KU201354 | KU201362 |
| Cali_145 | MF448248 | MF461422 | MF466166 | MF466173 |
| Cali_151 | MF448250 | MF461423 | MF466167 | ND |
| Cali_130 | MF448246 | MF461421 | MF466165 | ND |
| Cali_153 | MF448251 | MF461424 | MF466168 | ND |
| Cali_84 | KU341837 | KU171109 | KU201350 | MF466172 |
| Cali_103 | MF448243 | KU171111 | KU201352 | KU201360 |
| Cali_123 | MF448244 | KU171112 | KU201353 | KU201361 |
| Cali_143 | MF448247 | KU171115 | KU201356 | KU201364 |
| Cali_146 | MF448249 | KU171116 | KU201357 | KU201365 |
| Cali_156 | MF448252 | MF461425 | MF466169 | MF466174 |
| Cali_164 | MF448253 | MF461426 | MF466170 | MF466175 |
| Cali_167 | MF448254 | MF461427 | MF466171 | ND |
| SF_6 | MF461415 | KU230390 | KU238879 | KU238885 |
| SF_7 | MF461416 | KU230391 | KU238880 | KU238886 |
| SF_40 | MF461417 | KU230392 | KU238881 | KU238887 |
| SF_46 | MF461418 | KU230393 | KU238882 | KU238888 |
| SF_50 | MF461419 | KU230394 | KU238883 | KU238889 |
| SF_58 | MF461420 | KU230395 | KU238884 | KU238890 |
| CZ_178zB | MG986897 | MF503107 | MF503124 | MF503129 |
| CZ_177zB | MG986898 | MF503108 | MF503123 | MF503133 |
| CZ_192Z | MF503135 | MF503109 | MF503117 | MF503130 |
| CZ_3218 | MF503137 | MF503110 | MF503118 | MF503131 |
| CZ_4535 | MF503138 | MF503111 | MF503116 | MF503125 |
| CZ_190Z | MF503134 | MF503112 | MF503119 | MF503128 |
| CZ_PP1979B | MF503139 | MF503113 | MF503121 | MF503126 |
| CZ_S1120 | MF503140 | MF503114 | MF503120 | MF503132 |
| CZ_351 | MF503136 | MF503115 | MF503122 | MF503127 |

^1^ND, not determined
