# Supplementary material for: Sequence Variation of Rare Outer Membrane Protein β-Barrel Domains in Clinical Strains Provides Insights into the Evolution of Treponema pallidum subsp. pallidum, the Syphilis Spirochete
Source: mBio. 2018 Jun 12;9(3):e01006-18. doi: 10.1128/mBio.01006-18 (PMC6016234; doi:10.1128/mBio.01006-18)
Supplement: TEXT S1 [file mbo003183920s1.docx]

**Supplemental Material References**

1. **Marra C, Sahi S, Tantalo L, Godornes C, Reid T, Behets F, Rompalo A, Klausner JD, Yin Y, Mulcahy F, Golden MR, Centurion-Lara A, Lukehart SA.** 2010. Enhanced molecular typing of *Treponema pallidum*: geographical distribution of strain types and association with neurosyphilis. J Infect Dis **202:**1380-1388.

2. **Grillova L, Petrosova H, Mikalova L, Strnadel R, Dastychova E, Kuklova I, Kojanova M, Kreidlova M, Vanousova D, Hercogova J, Prochazka P, Zakoucka H, Krchnakova A, Vasku V, Smajs D.** 2014. Molecular typing of *Treponema pallidum* in the Czech Republic during 2011 to 2013: increased prevalence of identified genotypes and of isolates with macrolide resistance. J Clin Microbiol **52:**3693-3700.

3. **Tian H, Li Z, Li Z, Hou J, Zheng R, Li F, Liu R, Liu B, Wang C, Zhang F.** 2014. Molecular typing of *Treponema pallidum*: identification of a new sequence of *tp0548* gene in Shandong, China. Sex Transm Dis **41:**551.

4. **Read P, Tagg KA, Jeoffreys N, Guy RJ, Gilbert GL, Donovan B.** 2016. *Treponema pallidum* strain types and association with macrolide resistance in Sydney, Australia: new TP0548 gene types identified. J Clin Microbiol **54:**2172-2174.

5. **Mikalova L, Grillova L, Osbak K, Strouhal M, Kenyon C, Crucitti T, Smajs D.** 2017. Molecular typing of syphilis-causing strains among human immunodeficiency virus-positive patients in Antwerp, Belgium. Sex Transm Dis **44:**376-379.

6. **Grange PA, Allix-Beguec C, Chanal J, Benhaddou N, Gerhardt P, Morini JP, Deleuze J, Lassau F, Janier M, Dupin N.** 2013. Molecular subtyping of *Treponema pallidum* in Paris, France. Sex Transm Dis **40:**641-644.

7. **Mikalova L, Strouhal M, Oppelt J, Grange PA, Janier M, Benhaddou N, Dupin N, Smajs D.** 2017. Human *Treponema pallidum* 11q/j isolate belongs to subsp. *endemicum* but contains two loci with a sequence in TP0548 and TP0488 similar to subsp. *pertenue* and subsp. *pallidum*, respectively. PLoS Negl Trop Dis **11:**e0005434.

8. **Knauf S, Raphael J, Mitja O, Lejora IA, Chuma IS, Batamuzi EK, Keyyu JD, Fyumagwa R, Luert S, Godornes C, Liu H, Schwarz C, Smajs D, Grange P, Zinner D, Roos C, Lukehart SA.** 2016. Isolation of *Treponema* DNA from necrophagous flies in a natural ecosystem. EBioMedicine **11:**85-90.

9. **Li Z, Wang C, Xiao H, Zhao W, Li Z, Zheng R, Hou J, Huang N, Tian H.** 2017. Enhanced molecular typing of *Treponema pallidum* identified a new *tp0548* gene type in Shandong, China. APMIS **125:**937-939.

10. **Godornes C, Giacani L, Barry AE, Mitja O, Lukehart SA.** 2017. Development of a multilocus sequence typing (MLST) scheme for *Treponema pallidum* subsp. *pertenue*: application to yaws in Lihir Island, Papua New Guinea. PLoS Negl Trop Dis **11:**e0006113.

11. **Zobanikova M, Strouhal M, Mikalova L, Cejkova D, Ambrozova L, Pospisilova P, Fulton LL, Chen L, Sodergren E, Weinstock GM, Smajs D.** 2013. Whole genome sequence of the *Treponema* Fribourg-Blanc: unspecified simian isolate is highly similar to the yaws subspecies. PLoS Negl Trop Dis **7:**e2172.
